# Quantitative myelin-related maps from R1 and T2* ratio images using a single ME-MP2RAGE sequence in 7T MRI

**DOI:** 10.3389/fnana.2022.950650

**Published:** 2022-08-25

**Authors:** Jeong-Min Shim, Seo-Eun Cho, Seung-Gul Kang, Chang-Ki Kang

**Affiliations:** ^1^Neuroscience Research Institute, Gachon University, Incheon, South Korea; ^2^Department of Psychiatry, Gil Medical Center, Gachon University College of Medicine, Incheon, South Korea; ^3^Department of Radiological Science, College of Health Science, Gachon University, Incheon, South Korea

**Keywords:** myelin-related map, relaxation times, ratio mapping, ME-MP2RAGE, 7T MRI

## Abstract

**Background:** There still are limitations associated with quantifying myelin content using brain magnetic resonance imaging (MRI) despite several studies conducted on this subject. Therefore, this study aimed: (1) to propose a myelin-related mapping technique to obtain the quantitative R1/T2* (q-Ratio) that has the advantage of quick processing and less dependency on imaging parameters, (2) to validate this adapted q-Ratio method by comparing the quantitative myelin-related map with those acquired through an existing mapping method [T1-weighted/T2*-weighted (w-Ratio)], and (3) to determine the q-Ratio myelin-related values in the white and gray matter, and the relationship between the q-Ratio myelin-related value and cerebral volume size in regions of interest (ROIs) in a healthy population.

**Methods:** The multi-echo magnetization-prepared 2 rapid gradient echoes (ME-MP2RAGE) sequence was used in a 7 Tesla (7T) MRI for the acquisition of data regarding myelin content in 10 healthy participants. A correlation analysis was performed between myelin-related values obtained through the q-Ratio and w-Ratio methods. Additionally, myelin distribution was analyzed and compared in the white and gray matter, and the correlation between cerebral volume size and q-Ratio myelin-related value was analyzed in ROIs in the brain.

**Results:** The myelin-related maps acquired through the q-Ratio and w-Ratio methods were significantly correlated (*p* < 0.001), but the q-Ratio myelin-related map was much clearer. Additionally, the cerebral volume size in the gray matter was 399.40% larger than that in the white matter, but the q-Ratio myelin-related value in the gray matter was 80.83% lower than that of the white matter. Furthermore, volume size was positively correlated with q-Ratio myelin-related values in the white matter (*r* = 0.509, *p* = 0.006) but not in the gray matter (*r* = -0.133, *p* = 0.402).

**Conclusions:** In this study, we validated using a q-Ratio myelin-related map that was acquired in one imaging sequence at 7T MRI. In addition, we found a significant correlation between ROI volume size and the q-Ratio myelin-related value in the white matter but not in the gray matter. It is expected that this technique could be applied to the study of various neuropsychiatric diseases related to demyelination in the future.

## Introduction

Myelin surrounds the axon of neurons and is an essential component for maintaining efficient brain function (Ganzetti et al., 2014). Myelin maintains nerve fiber integrity and speeds up the propagation of action potentials, thereby facilitating proper brain function (Salzer, 2015). When myelin is damaged, it interferes with the normal signal transmission of nerves, which causes various symptoms affecting sensory function, cognition, and behavior (Papuć and Rejdak, 2018). Therefore, it is considered important to conduct an accurate quantitative evaluation of myelin in neuropsychiatric disorders, such as multiple sclerosis (Love, 2006), neuromyelitis optica spectrum disorder (Jeong et al., 2017), traumatic brain injury (Mierzwa et al., 2015; Shi et al., 2015), Alzheimer’s disease (Fornari et al., 2012), and major depressive disorder (Sacchet and Gotlib, 2017).

Recently, based on research results indicating that myelin as well as its surrounding lipids, water, and iron affect longitudinal (T1 or R1) and transverse (T2 and T2*) relaxation times, several studies on noninvasive myelin evaluation methods using magnetic resonance imaging (MRI) have been initiated (Koenig, 1991; Miot-Noirault et al., 1997; Schmierer et al., 2004; Callaghan et al., 2015; Haast et al., 2016). Callaghan et al. studied the dependence of R1 on local microstructural tissue properties, such as macromolecular characteristics, iron content, and water content (Callaghan et al., 2015). Miot-Noirault et al. (1997) also visualized gray matter differentiation on MRI using the T2 relaxation time, and they quantitatively analyzed the MRI data in order to evaluate the progress of myelination in the brain. However, these studies only used data derived from one of the various types of contrast images. Therefore, several studies have pursued methods involving the use of multiple contrast images and/or various imaging variables, which could possibly aid in acquiring improved myelin maps. Ganzetti et al. (2014) used the T1-weighted/T2-weighted technique for myelin mapping, and Haast et al. compared various methods for obtaining a myelin-related image suitable for subject-specific anatomical cortical parcellation (Haast et al., 2016). As such, cerebral myelination has been studied with various techniques that provide the contrast-enhanced images, such as T1-weighted/T2*-weighted (w-Ratio), R1, and T2* methods. Consequently, it was found that high signal intensity in cortical regions with high myelination could be seen in T1-weighted and R1 images, and low signal intensity, in T1 and T2* images (Sigalovsky et al., 2006; Geyer et al., 2011; Glasser and Van Essen, 2011; Cohen-Adad et al., 2012; Dick et al., 2012; Bock et al., 2013; Sereno et al., 2013; Lutti et al., 2014; De Martino et al., 2015; Haast et al., 2016).

Among the myelin evaluation methods, methods involving the division of T1-weighted images by T2*-weighted images resulted in an improvement in myelin contrast between heavily myelinated and non-myelinated regions (Glasser and Van Essen, 2011; De Martino et al., 2015) and in the effective removal of receive bias field owing to image division (Haast et al., 2016). However, there are limitations associated with weighted images, as they are not quantitative and stable values corresponding to the basic biochemical composition of tissues; this is because these images vary according to MRI imaging parameters and the selected sequences. On the other hand, the myelin evaluation method using R1 and T2* has quantitative value; however, the acquisition time (TA) is long because a large number of acquisition datasets are required for avoiding systematic bias in the MRI parameter estimates. In addition, since systematic bias is usually larger than the subtle changes in the MRI signal resulting from myelin content changes, it should be appropriately controlled in order to obtain quantitative MRI parameter estimates of the tissue microarchitecture (Lutti et al., 2014). Based on this, many attempts have been made to obtain quantitative and more reliable values in order to improve the contrast of myelinated areas by determining the myelin content through the R1/T2* (q-Ratio) method (Haast et al., 2016). However, these previous attempts have required a long TA due to multiple image sequences as well as an additional process for the registration of the pixel positions in images obtained *via* different sequences.

Therefore, in this study, we aimed: (1) to introduce a technique to obtain a q-Ratio myelin-related map that has the advantage of quick processing and less dependency on imaging parameters, (2) to validate this method by comparing the acquired q-Ratio myelin-related maps with those acquired through the existing mapping method (w-Ratio), and (3) to determine the q-Ratio myelin-related values in white and gray matter and the relationship between the q-Ratio myelin-related value and the volume size of brain regions of interest (ROIs) in a healthy population.

## Materials and Methods

### Subjects

Ten healthy volunteers (male:female = 2:8) aged 20–65 years (mean ± standard deviation: 32.7 ± 11.8) participated in this study. They were recruited as healthy controls in relation to patients with major depressive disorder. A board-certified psychiatrist interviewed all the participants and assessed their eligibility for participation in this study using a structured clinical interview employing the fifth edition of the Diagnostic and Statistical Manual of Mental Disorders (DSM-5; First et al., 2015). The following exclusion criteria were applied: left-handedness according to the Edinburgh Handedness Test (Oldfield, 1971), unstable or major medical condition, neurological disorders, any psychiatric history, history of taking psychotropic medications, substance use disorder, intellectual disability, personality disorder, first-degree relatives with major psychiatric disorders, neurocognitive disorders, history of head trauma, previous abnormal findings on brain imaging, contraindications to MRI (e.g., metals in the body), and pregnancy or lactation. This study was approved by the Institutional Review Board of Gil Medical Center (IRB No. GDIRB2018-005); further, before starting the experiment, the purpose of the study was explained to all the participants, and written informed consent was obtained.

### Data acquisition parameters

Sagittal images of the whole brain were obtained with an 8-channel phase-array coil using a 7T MRI system (Magnetom, Siemens, Erlangen, Germany). In order to ensure that the participants were in a comfortable position, a pillow was placed under the head, and a foam cushion was used to minimize head movement. The prototype multi-echo magnetization-prepared 2 rapid gradient echoes (ME-MP2RAGE) sequence was used (Metere et al., 2017), and images of 208 slices were obtained with repetition time (TR) of 8,000 ms; echo times (TEs) of 3.46, 7.28, 11.1, and 14.92 ms; inversion times (TI) of 1,000 and 3,200 ms; generalized auto-calibrating partially parallel acquisitions (GRAPPA) with accelerating factor = 3 (50 reference lines); 7/8 and 6/8 partial Fourier factors along the phase-encoding (PE) and slice-encoding (SE) directions, respectively; and a matrix size of 256 × 256. The acquisition parameters used in this study are shown in Table 1.

**Table 1:** Acquisition parameters used with the ME-MP2RAGE sequence in 7T MRI.

	**MR parameters for the ME-MP2RAGE sequence**
TR (ms)	8,000
TEs (ms)	3.46, 7.28, 11.1, 14.92
TI 1/TI 2 (ms)	1,000/3,200
FAs (°)	4/4
Accelerating factor (GRAPPA)	3
Reference lines	50
Phase partial Fourier	7/8
Slice partial Fourier	6/8
FOV (mm^3^)	166 × 166 × 135.2
Imaging resolution (mm^3^)	0.65
Matrix size	256 × 256
Number of slices	208
Bandwidth (Hz/Px)	280
TA	14 min 16 s

Abbreviations: 7T MRI, 7 Tesla magnetic resonance imaging; FA, flip angle; FOV, field-of-view; GRAPPA, generalized auto-calibrating partially parallel acquisitions; ME-MP2RAGE, multi-echo magnetization-prepared 2 rapid gradient echoes; TA, acquisition time; TE, echo time; TI, inversion time; TR, repetition time.

### Image processing

Both T1 and T2* maps were created from the images acquired in the single sequence, but they were created through separate processes. T1 maps were generated immediately after data acquisition as part of the built-in data acquisition process. T2* maps, however, were generated through a separate process that required fitting an appropriate algorithm to the acquired images. The University of Edinburgh’s “Fit T2 or T2 Star MRI data and create parameters maps”^1^ tool was employed to generate R2* maps with multiple echo images created from the second inversion radiofrequency pulse, which involved the use of the non-linear least-square curve fitting method with the Levenberg–Marquardt algorithm (Thrippleton et al., 2019). For the verification of the q-Ratio technique, the first TE (TE1) image of the first TI (TI1) process and fourth TE (TE4) image of the second TI (TI2) process were used as the T1-weighted and T2*-weighted images, respectively.

For the group analysis of cerebral myelin-related maps, T1-weighted, T2*-weighted, T1, and R2* images were normalized to the Montreal Neurological Institute (MNI) 152 template in the standard space using SPM12 by performing the following steps. For easy segmentation, the T1 map was contrast-inverted, segmented and divided into gray matter, white matter, and cerebrospinal fluid (CSF). A mask image was created by adding gray matter, white matter, and bone images and was multiplied by an inverted T1 map to generate an image aligned to the MNI template. T1-weighted images, T2*-weighted images, original T1 maps, and R2* maps were added to the “images to write” and were normalized with a voxel size of 0.5 mm.

**Figure 1:**
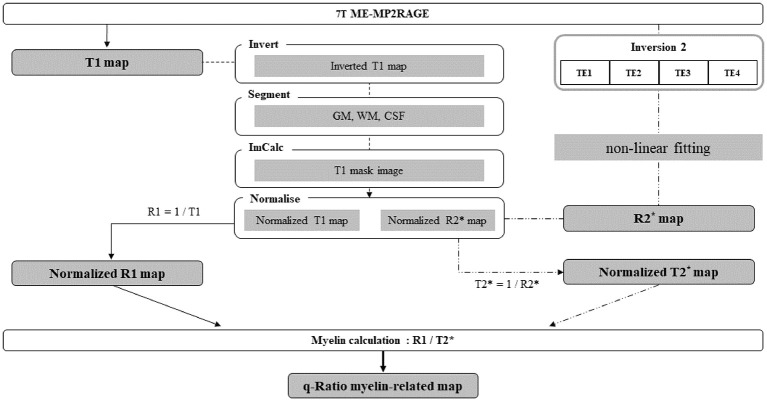
Schematic diagram of the entire procedure from image acquisition to myelin-related mapping.

**Figure 2:**
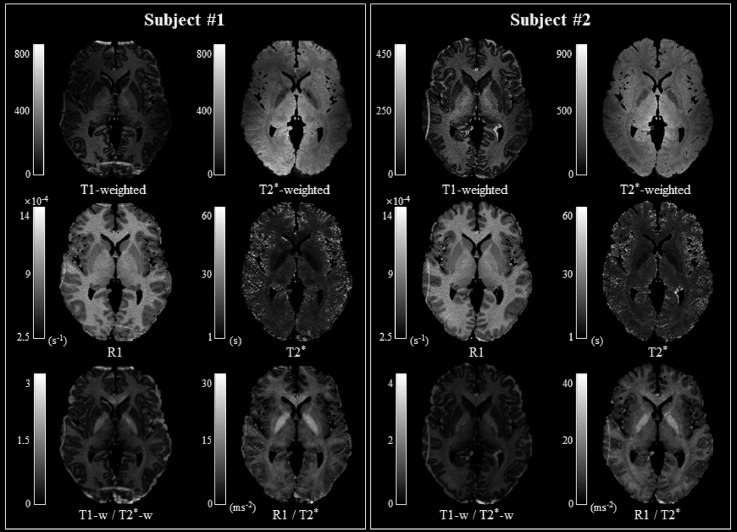
Comparison of myelin-related mapping methods. The myelin-related maps obtained with the T1-weighted/T2*-weighted (w-Ratio) and R1/T2* (q-Ratio) methods are similar in both subjects. Note that the FOV was not optimized during image acquisition and that aliasing artifacts occurred, depending on the subjects’ head sizes, mainly along the anterior and posterior regions in Subject 1 and the left and right regions in Subject 2. Abbreviations: FOV, field-of-view.

A T2* map (e.g., 1/R2* map) was created from the normalized R2* map, which was registered in the standard space. The T1 and T2* ranges were set to 700–4,000 ms and 1–60 ms, respectively (Metere et al., 2017). A q-Ratio myelin-related map was created by dividing the R1 map by the T2* map (Figure 1). In addition, to verify the q-Ratio technique, a w-Ratio myelin-related map was also created by dividing the normalized T1-weighted image by the T2*-weighted image (Figure 2). As artifacts around the edge of the brain can make further analysis difficult (visible in Figure 2), we set the thresholds of the gray and white matter to 0.4 and 0.6, respectively, to remove the artifactual signals in the w-Ratio myelin-related map. Furthermore, the voxels in which the artifacts were removed in the w-Ratio map were used as a mask image for obtaining the q-Ratio myelin-related values.

### Validation and analysis of the adapted q-Ratio technique

To validate the q-Ratio myelin-related mapping technique, the q-Ratio myelin-related map was compared with the w-Ratio myelin-related map. First, to assess the sensitivity to myelin-related variation, the difference between averaged myelin-related values in the white and gray matter in both q-Ratio and w-ratio myelin-related maps was compared. Additionally, the inter-subject coefficient of variation (COV) was calculated for both the q-Ratio and w-Ratio maps to examine the myelination variability across subjects (Brown, 1998). As Haast et al. (2016) found that R1 myelin-related maps had lower inter-subject COV than did either q-Ratio (obtained through two sequences) or w-Ratio myelin-related maps, R1 myelin-related maps were also included in the COV analysis for comparison.

**Figure 3:**
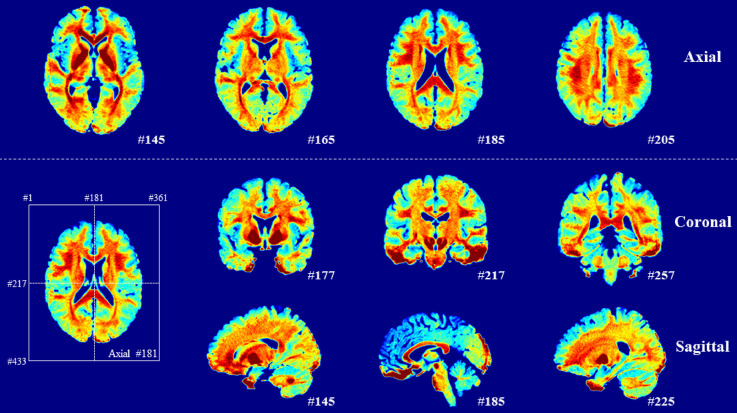
Averaged myelin-related maps of total participants. A high signal appears in the white matter.

For the selection of ROIs in the gray and white matter in the left hemisphere (Ganzetti et al., 2014), we used the Johns Hopkins University (JHU) ICBM-DTI-81 white matter labels atlas (Mori et al., 2008) and Automated Anatomical Labeling atlas 3 (AAL3; Rolls et al., 2020), which were also normalized to 0.5 mm. For further comparison, five representative ROIs were selected in the gray matter [superior frontal gyrus-dorsolate (Frontal_Sup_2), lingual gyrus (Lingual), middle occipital gyrus (Occipital_Mid), superior temporal gyrus (Temporal_Sup), and anterior cingulate cortex (subgenual; ACC_sub)], and seven representative ROIs were selected in the white matter [genu of corpus callosum (GCC), anterior limb of internal capsules (ALIC), posterior limb of internal capsules (PLIC), retrolenticular limb of internal capsules (RLIC), anterior corona radiata (ACR), posterior thalamic radiation (including optic radiation; PTR), and external capsule (EC)]. To examine the cerebral myelin distribution, the average myelin-related map of 10 subjects was obtained (Figure 3).

For this comparison, the subcortical and cerebellar areas were excluded to facilitate clear differentiation between white and gray matter because these areas usually include more complicated neural components and a relatively large distribution of nerve fibers. Therefore, the gray matter included the cerebral and cingulate cortices allocated with AAL3. Regardless of gray or white matter, the w-Ratio and q-Ratio myelin-related maps within all the selected ROIs were analyzed using the correlation analysis. Furthermore, the correlation between q-Ratio myelin-related value and cerebral volume size in each ROI in the gray and white matter was analyzed using the data of the 10 subjects. This was performed using MATLAB 2018b (The MathWorks Inc., Natick, MA, USA).

## Results

The inter-subject COVs for the q-Ratio, w-Ratio, and R1 maps in the white matter were 0.321 (R1) < 0.556 (q-Ratio) < 0.644 (w-Ratio). In the gray matter, COVs were 0.521 (R1) < 0.839 (q-Ratio) < 0.911 (w-Ratio).

The q-Ratio myelin-related map in two representative subjects was comparable to the w-Ratio myelin-related map, as shown in Figure 2. In the w-Ratio myelin-related map, exaggerated signals were found at the edges of the brain. On the other hand, in the q-Ratio myelin-related map, the intensity of these signals was substantially decreased.

The contrast differences between the white and gray matter were 44.67% (*z* = 0.523) and 53.50% (*z* = 0.547) in the q-Ratio and the w-Ratio maps, respectively. The difference between the white and gray matter was much clearer when the myelin-related maps from 10 subjects were averaged (Figure 3). However, when TI2-TE3 was used instead of TI2-TE4, the contrast difference between the white and gray matter decreased to 46.93% (*z* = 0.536) for the w-Ratio map.

**Figure 4:**
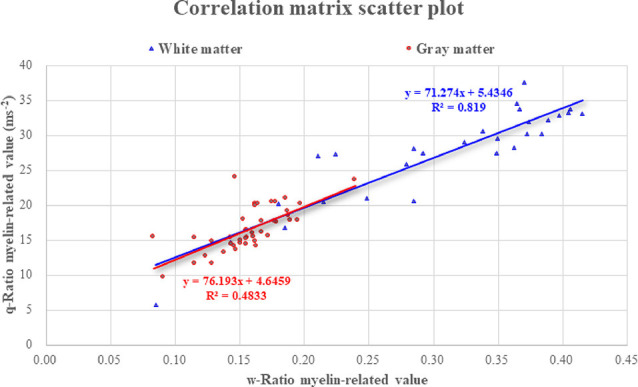
Correlation between the two myelin-related mapping methods. Abbreviations: ROI, region of interest.

The average of myelin-related values calculated using the w-Ratio method was 0.307 in the white matter and 0.157 in the gray matter. When TE3 images of TI2 were used as the T2*-weighted images, the averaged w-Ratio myelin-related values were 0.266 in the white matter and 0.165 in the gray matter. The average of myelin-related values calculated using the q-Ratio method was 27.31 ms^−2^ in the white matter and 16.62 ms^−2^ in the gray matter. In the correlation analysis between the myelin-related values obtained through the two mapping methods, there was a significant positive correlation in both the gray and white matter (*r* = 0.706, *p* < 0.001; *r* = 0.899, *p* < 0.001, respectively; Figure 4).

**Figure 5:**
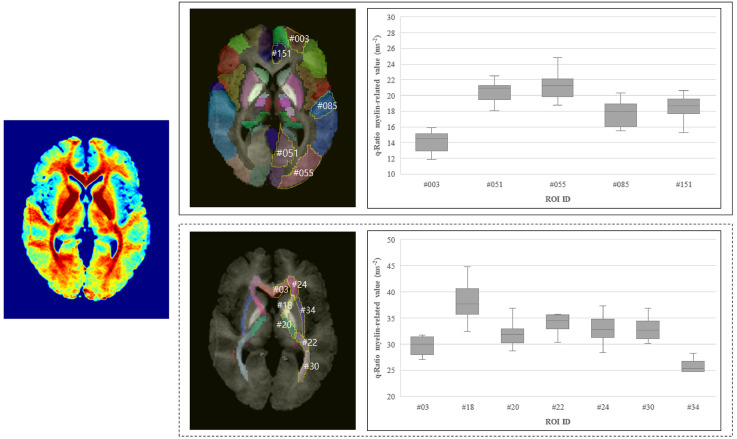
Q-Ratio myelin-related values in the representative ROIs in the gray and white matter in the left hemisphere. Averaged myelin-related map of 10 subjects was shown on the left. Q-Ratio myelin-related values in the gray matter (right-top) and in the white matter (right-bottom) were compared. Abbreviations: ROIs, regions of interest. In the gray matter, #003: Frontal_Sup_2 (superior frontal gyrus, dorsolateral), #051: Lingual (lingual gyrus), #055: Occipital_Mid (middle occipital gyrus), #085: Temporal_Sup (superior temporal gyrus), #151: ACC_sub (anterior cingulate cortex, subgenual). In the white matter, #03: GCC (genu of corpus callosum), #18: ALIC (anterior limb of internal capsule), #20: PLIC (posterior limb of internal capsule), #22: RLIC (retrolenticular part of internal capsule), #24: ACR (anterior corona radiate), #30: PTR [posterior thalamic radiation (include optic radiation)], #34: EC (external capsule).

In the selected ROIs in the left hemisphere of the participants, the q-Ratio myelin-related values in the white matter were higher (range: 21.70–44.85 ms^−2^) than those in the gray matter (range: 11.81–24.81 ms^−2^; Figure 5). The q-Ratio myelin-related values in the selected representative ROIs in Figure 5 were as follows: GCC in the white matter area, 30.24 ms^−2^; ALIC, 37.94 ms^−2^; PLIC, 31.95 ms^−2^; RLIC, 34.09 ms^−2^; ACR, 32.89 ms^−2^; PTR, 32.90 ms^−2^; and EC, 25.49 ms^−2^. The q-Ratio myelin-related values in Frontal_Sup2 in the gray matter area was 14.17 ms^−2^ and those in the Lingual, Occipital_Mid, TemporalSup, and ACCsub cortices were 20.48, 21.23, 17.73, and 18.42 ms^−2^, respectively.

**Figure 6:**
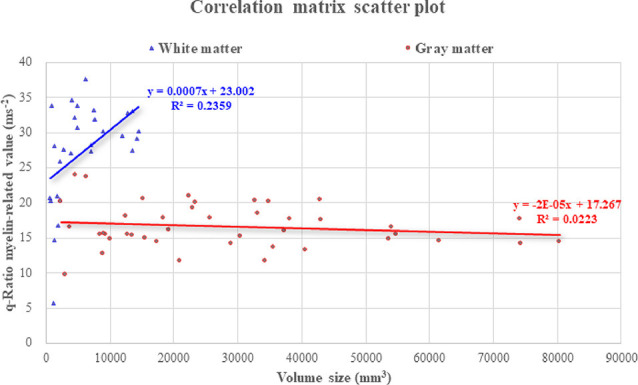
Correlation of q-Ratio myelin-related value and cerebral volume size in the gray and white matter. In the white matter, there was a significant correlation between the cerebral volume size and myelin-related value (*r* = 0.509, *p* = 0.006), and the slope of the trend line was observed to increase more steeply. However, there was no significant relationship with cerebral volume size in the gray matter.

The results corresponding to the cerebral volume size and myelin-related value derived through the q-Ratio method were as follows. The median [95% confidence interval (CI)] q-Ratio myelin-related value in the white matter and cerebral volume size were 28.68 (24.70–29.93) ms^−2^ and 4,653.26 (4,126.84–7,574.65) mm^3^, respectively (Table 2), and the median q-Ratio myelin-related value in the gray matter (cortical area) and cerebral volume size were 15.86 (15.68–17.57) ms^−2^ and 23,238.44 (22,155.09–34,566.04) mm^3^, respectively (Table 3). Although the volume size of the gray matter was 399.40% larger than that of the white matter, the q-Ratio myelin-related value of the white matter was 80.83% higher than that of the gray matter. Table 2 presents the q-Ratio myelin-related value and cerebral volume size in each ROI in the white matter; there was a positive correlation between the cerebral volume size and q-Ratio myelin-related value (*r* = 0.509, *p* = 0.006). On the other hand, there was no significant correlation in the gray matter (*r* = -0.133, *p* = 0.402; Figure 6).

**Table 2:** Quantitative ratio (q-ratio) myelin-related values and cerebral volume sizes in the white matter ROIs.

**ROI**	**Volume size (mm^3^)**	**Myelin-related value (ms^−2^):Mean (±SEM)**	**ROI**	**Volume size (mm^3^)**	**Myelin-related value (ms^−2^):Mean (±SEM)**
MCP	1,4230.88	29.12 (±0.66)	ACR	13,455.88	33.11 (±0.82)
PCT	1,377.50	28.18 (±0.68)	SCR	14,489.25	30.24 (±0.51)
GCC	8,847.50	30.24 (±0.90)	PCR	7,095.25	28.24 (±0.51)
BCC	13,535.25	27.43 (±0.60)	PTR	7,515.13	33.27 (±0.89)
SCC	11,914.13	29.56 (±0.76)	SS	4,458.13	32.22 (±0.65)
FX	635.50	20.71 (±0.82)	EC	7,091.25	27.30 (±0.55)
CST	2,727.63	27.55 (±0.62)	CGC	4,926.25	30.70 (±0.78)
ML	1,296.75	14.76 (±0.53)	PHC	2,164.88	20.51 (±0.48)
ICP	1,751.50	21.03 (±0.42)	FX-ST	2,204.13	25.93 (±0.83)
SCP	1,848.38	16.78 (±0.32)	SLF	12,748.88	32.85 (±0.59)
CP	4,108.75	34.66 (±1.13)	SFO	893.38	33.81 (±0.80)
ALIC	6,250.50	37.63 (±1.13)	IFO	3,927.13	27.10 (±0.66)
PLIC	7,590.25	31.96 (±0.63)	UF	739.88	20.26 (±0.81)
RLIC	4,848.38	33.83 (±0.51)	Tapetum	1,148.50	5.72 (±0.46)
Median volume size (95% CI)			4,653.26 (4,126.84–7,574.65)		
Median myelin-related value (95% CI)			28.68 (24.70–29.93)		

Abbreviations: ACR, anterior corona radiata; ALIC, anterior limb of internal capsule; BCC, body of corpus callosum; CGC, cingulum (cingulate gyrus); CI, confidence interval; CP, cerebral peduncle; CST, corticospinal tract; EC, external capsule; FX, fornix (column and body of fornix); FX-ST, fornix (cres)/stria terminalis; GCC, genu of corpus callosum; ICP, inferior cerebellar peduncle; IFO, inferior fronto-occipital fasciculus; MCP, middle cerebellar peduncle; ML, medial lemniscus; PCR, posterior corona radiata; PCT, pontine crossing tract (a part of MCP); PHC, cingulum (hippocampus); PLIC, posterior limb of internal capsule; PTR, posterior thalamic radiation (including optic radiation); RLIC, retrolenticular part of internal capsule; SCC, splenium of corpus callosum; SCP, superior cerebellar peduncle; SCR, superior corona radiata; SFO, superior fronto-occipital fasciculus; SLF, superior longitudinal fasciculus; SS, sagittal stratum (including inferior longitudinal fasciculus and IFO); UF, uncinate fasciculus.

**Table 3:** Quantitative ratio (q-ratio) myelin-related values and cerebral volume sizes in the gray matter ROIs.

**ROI**	**Volume size (mm^3^)**	**Myelin-related value (ms^−2^):Mean (±SEM)**	**ROI**	**Volume size (mm^3^)**	**Myelin-related value (ms^−2^):Mean (±SEM)**
Precentral	54,857.00	15.53 (±0.42)	Lingual	34,800.25	20.23 (±0.43)
Frontal_Sup_2	80,349.38	14.49 (±0.41)	Occipital_Sup	22,327.88	20.99 (±0.54)
Frontal_Mid_2	74,327.50	14.48 (±0.30)	Occipital_Mid	42,855.88	20.49 (±0.46)
Frontal_Inf_Oper	19,282.00	16.17 (±0.46)	Occipital_Inf	15,224.88	20.54 (±0.52)
Frontal_Inf_Tri	37,257.63	16.08 (±0.21)	Fusiform	38,190.88	17.71 (±0.42)
Frontal_Inf_Orb_2	13,489.88	15.41 (±0.33)	Postcentral	61,599.63	14.61 (±0.39)
Rolandic_Oper	18,416.50	17.86 (±0.41)	Parietal_Sup	34,309.13	11.76 (±0.43)
Supp_Motor_Area	35,649.25	13.72 (±0.45)	Parietal_Inf	30,402.50	15.26 (±0.44)
Olfactory	4,562.25	24.02 (±0.88)	SupraMarginal	25,711.00	17.87 (±0.35)
Frontal_Sup_Medial	40,595.75	13.24 (±0.46)	Angular	23,036.25	19.21 (±0.48)
Frontal_Med_Orb	12,520.38	18.08 (±0.72)	Precuneus	54,041.63	16.51 (±0.45)
Rectus	12,818.75	15.49 (±1.11)	Paracentral_Lobule	17,350.25	14.43 (±0.48)
OFCmed	9,399.75	15.45 (±0.81)	Heschl	3,740.13	16.50 (±0.41)
OFCant	8,553.13	15.45 (±0.45)	Temporal_Sup	43,110.75	17.64 (±0.36)
OFCpost	8,925.50	12.79 (±0.56)	Temporal_Pole_Sup	20,928.25	11.76 (±0.31)
OFClat	3,086.25	9.72 (±0.39)	Temporal_Mid	74,119.88	17.70 (±0.26)
Insula	29,061.88	14.26 (±0.31)	Temporal_Pole_Mid	15,475.75	15.00 (±0.49)
Cingulate_Mid	33,138.50	18.53 (±0.34)	Temporal_Inf	53,663.75	14.85 (±0.59)
Cingulate_Post	6,293.63	23.66 (±0.55)	ACC_sub	2,391.88	20.25 (±0.62)
Calcarine	32,723.25	20.29 (±0.47)	ACC_pre	10,083.00	14.84 (±0.47)
Cuneus	23,440.63	20.02 (±0.41)	ACC_sup	9,031.50	15.64 (±0.58)
Median volume size (95% CI)			23,238.44 (22,155.09–34,566.04)		
Median myelin-related value (95% CI)			15.86 (15.68–17.57)		

Abbreviations: ACC_pre, anterior cingulate cortex (pregenual); ACCsub, anterior cingulate cortex (subgenual); ACCsup, anterior cingulate cortex (supracallosal); Angular, angular gyrus; Calcarine, calcarine fissure and surrounding cortex; CI, confidence interval; CingulateMid, middle cingulate and paracingulate gyri; CingulatePost, posterior cingulate; Cuneus, cuneus; FrontalInfOper, inferior frontal gyrus (opercular part); FrontalInfOrb2, inferior frontal gyrus pars orbit; FrontalInfTri, inferior frontal gyrus (triangular part); FrontalMedOrb, superior frontal gyrus (medial orbital); FrontalMid2, middle frontal gyrus; FrontalSup2, superior frontal gyrus (dorsolate); FrontalSupMedial, superior frontal gyrus (medial); Fusiform, fusiform gyrus; Heschl, Heschl’s gyrus; Insula, insula; Lingual, lingual gyrus; OccipitalInf, inferior occipital gyrus; OccipitalMid, middle occipital gyrus; OccipitalSup, superior occipital gyrus; OFCant, anterior orbital gyrus; OFClat, lateral orbital gyrus; OFCmed, medial orbital gyrus; OFCpost, posterior orbital gyrus; Olfactory, olfactory cortex; ParacentralLobule, paracentral lobule; ParietalInf, inferior parietal gyrus (excluding supramarginal and angular gyri); ParietalSup, superior parietal gyrus; Postcentral, postcentral gyrus; Precentral, precentral gyrus; Precuneus, precuneus; Rectus, gyrus rectus; RolandicOper, rolandic operculum; SuppMotorArea, supplementary motor area; SupraMarginal, supramarginal gyrus; TemporalInf, inferior temporal gyrus; TemporalMid, middle temporal gyrus; TemporalPoleMid, temporal pole (middle temporal gyrus); TemporalPoleSup, temporal pole (superior temporal gyrus); TemporalSup, superior temporal gyrus.

## Discussion

In this study of cerebral myelin mapping, high-resolution images were obtained within a reasonable acquisition time using the ME-MP2RAGE sequence at 7T MRI, and quantitative myelin-related mapping was performed using the q-Ratio method. The acquired myelin-related map was comparable to those created using weighted images, which have been studied in various ways, thus validating the reliability of the proposed method. In addition, the results of the comparison of the q-Ratio myelin-related values in the white and gray matter of healthy subjects suggest the possibility of further developing the proposed method for various clinical applications in the future.

Although the ME-MP2RAGE sequence used in this study had the advantage of obtaining multiple images with one imaging scan, the TA (14 min 16 s), while reasonable, might not seem to be advantageous compared to a typical acquisition collected over multiple shorter sequences. However, TA can be reduced by adjusting parameters that affect TA, such as TR and TI. Therefore, the proposed technique allows for effectively acquiring multiple images in a single sequence, providing simplicity in image processing while preserving the independence of image parameters and/or the possibility of shortening the TA.

Among the myelin-related mapping methods, R1 had the lowest COV. T1 (or R1) is known to be an MR parameter closely related to tissue myelination and myelin-bound cholesterol, and it is a contrast source in which myelin content dominates (Haast et al., 2016). However, oligodendrocytes, glial cells of the central nervous system that produce myelin, are known to stain iron more strongly than other cells in the normal adult brain and require a certain amount of iron for the production and maintenance of myelin (Todorich et al., 2009). As we age, especially when neurons begin to accumulate high levels of iron, oxidative stress-related neurodegeneration and myelin destruction can occur, which can lead to neurological disorders such as Alzheimer’s disease (Todorich et al., 2009). As such, myelin is considered important in neurological and psychiatric disorders. Moreover, pathologically, it is known that various neurodegenerative disorders and iron levels in the brain area are correlated (Berg and Youdim, 2006). Therefore, it is necessary to weight the iron contribution to better assess myelin information. The iron contribution in T1 maps (=1/R1 map) is, on average, 10% in the white matter and 36% in the gray matter (Stüber et al., 2014; Haast et al., 2016). In contrast, the T2* map (=1/R2* map) is known as a contrast source dominated by iron (Haast et al., 2016). Additionally, myelin studies using R2* and quantitative susceptibility mapping confirm that R2* is directly proportional to myelin-related content as well as iron content (Wood et al., 2005; Lee et al., 2017). Therefore, through this study, we suggest that the proposed method could aid in the interpretation of myelin in relation to iron, particularly in cases of aging and neurodegenerative or psychiatric disorders.

The verification of the q-Ratio technique and comparison with the w-Ratio myelin-related mapping technique revealed that both methods produced a similar pattern of showing a high signal in the white matter (Figure 2), which was seen much more clearly in the averaged myelin-related map (Figure 3). This qualitative observation was corroborated through an objective measure calculating the difference between gray and white matter, confirming that the w-Ratio myelin-related map was slightly more sensitive to the myelin-related variations than was the q-Ratio myelin-related map. Further, the myelin-related value calculated using the q-Ratio myelin-related mapping method was significantly correlated with that calculated using the w-Ratio myelin-related mapping method in both the gray and white matter, as shown in Figure 4. Considering that the R1 and T2* maps used in the q-Ratio method contain quantitative information, it appears that the adapted q-Ratio method is superior to the w-Ratio method. Therefore, it seems that the q-Ratio map proposed here with images acquired in one sequence is sufficient to replace the w-Ratio map.

Based on these results, it was confirmed that the limitations of weighted images obtained with various MRI parameters and of those collected with different MRI pulse sequences, which provide inconsistent myelin-related contrasts (Ganzetti et al., 2014), could be overcome with the myelin-related value calculated noninvasively and quantitatively using q-Ratio images. This is because weighted images cannot provide constant contrasts, as they vary with the MRI acquisition parameters, and it might be necessary to capture images with different sequences for further registration processing. For comparing the q-Ratio method with the w-Ratio method in this study, we used the TE1 image of TI1 as the T1-weighted image and the TE4 image of TI2 as the T2*-weighted image; however, the myelin-related value is likely to change when different weighted images are used during the w-Ratio mapping (e.g., TE2 image of TI1 and TE3 image of TI2). Indeed, when TI1-TE1 was divided by TI2-TE3, the w-Ratio values were 0.165 and 0.266 in the gray and white matter, respectively, while when divided by TI2-TE4, the w-Ratio values were 0.157 and 0.307 in the gray and white matter, respectively. Furthermore, when TI2-TE3 was used for the w-Ratio map, the difference between gray and white matter decreased, from 53.50% (*z* = 0.547) with TI2-TE4 to 46.93% (*z* = 0.536). Given that the w-Ratio value can vary depending on which sequence timing is used even with the same parameters, this variability reconfirms the limitations of the w-Ratio method. Therefore, it seems necessary to compare the myelin-related values obtained through our q-Ratio method with those obtained using other w-Ratio methods.

The results of the analysis of the q-Ratio myelin-related values in the white and gray matter ROIs in healthy subjects revealed that the average cerebral volume size in the white matter ROIs was smaller than that in the gray matter ROIs; however, the average q-Ratio myelin-related value in the white matter ROIs was higher. The median q-Ratio myelin-related value in the white matter ROIs was 28.68 ms^−2^ and that was 15.86 ms^−2^ in the gray matter ROIs. In addition to these results, the cerebral volume size and q-Ratio myelin-related value were positively correlated in the white matter, which is mainly composed of myelinated axons. However, there was no significant correlation in the gray matter, which is presumably because myelin is not a dominant component in the gray matter compared to in the white matter and because the gray matter also has other complicated components.

In conclusion, we were able to validate the quantitative measurement of myelin through the q-Ratio technique. Further, it was confirmed that the cerebral volume size and q-Ratio myelin-related value are positively correlated in the white matter, but this correlation was not seen in the gray matter. However, an in-depth study of the normal range of myelin was still not conducted due to the small number of participants. Therefore, in future studies, larger sample sizes must be used, and the effect of neuropsychiatric disorders on myelin must be examined by comparing the q-Ratio myelin-related values between healthy subjects and patients with neuropsychiatric disorders. In addition, due to the inhomogeneity of 7T MRI, q-Ratio myelin-related values in some sensitive regions still be carefully considered and should be studied further in larger samples. Furthermore, the stability of the q-Ratio method proposed here should be further examined through comparison of various sequences and further validation of various imaging parameters and analyses of test-retest data. In addition, the gray matter atlas includes white matter components within the gray matter; due to this potential limitation, it is necessary to perform an analysis according to the components of the gray and white matter within the ROIs in a future study. Finally, some regions in the subcortical areas, such as the thalamus, pallidum, and putamen, where the myelin-related signal was very high, should be further analyzed. If these efforts were undertaken in further studies, they can be expected to make a great contribution to the identification of the pathophysiology of neuropsychiatric disorders caused by demyelination.

## Data Availability Statement

The raw data supporting the conclusions of this article will be made available by the authors, without undue reservation.

## Ethics Statement

The studies involving human participants were reviewed and approved by the Institutional Review Board of Gil Medical Center (IRB No. GDIRB2018-005). The patients/participants provided their written informed consent to participate in this study.

## Author Contributions

J-MS, S-GK, and C-KK contributed to the conception and design of the study. S-EC and S-GK recruited the participants and conducted the experiments. J-MS and C-KK analyzed the data and wrote the first draft of the manuscript. J-MS, S-GK, and C-KK edited the manuscript. All the authors contributed to the article and approved the submitted version.

## Funding

This work was supported by the National Research Foundation of Korea (NRF) grant funded by the South Korean government (MSIT; NRF-2020R1A2C1004355, NRF-2020R1A2C1007527). This research was also funded by the Korea Health Technology R&D Project through the Korea Health Industry Development Institute, funded by the Ministry of Health and Welfare, South Korea (grant number: HI17C2665).

## Footnotes

^1^ http://github.com/mjt320/T2Star
